# Supplementary figures and images for: Age and remission induction therapy for acute myeloid leukemia: An analysis of data from the Korean acute myeloid leukemia registry
Source: PLoS One. 2021 May 7;16(5):e0251011. doi: 10.1371/journal.pone.0251011 (PMC8104390; doi:10.1371/journal.pone.0251011)

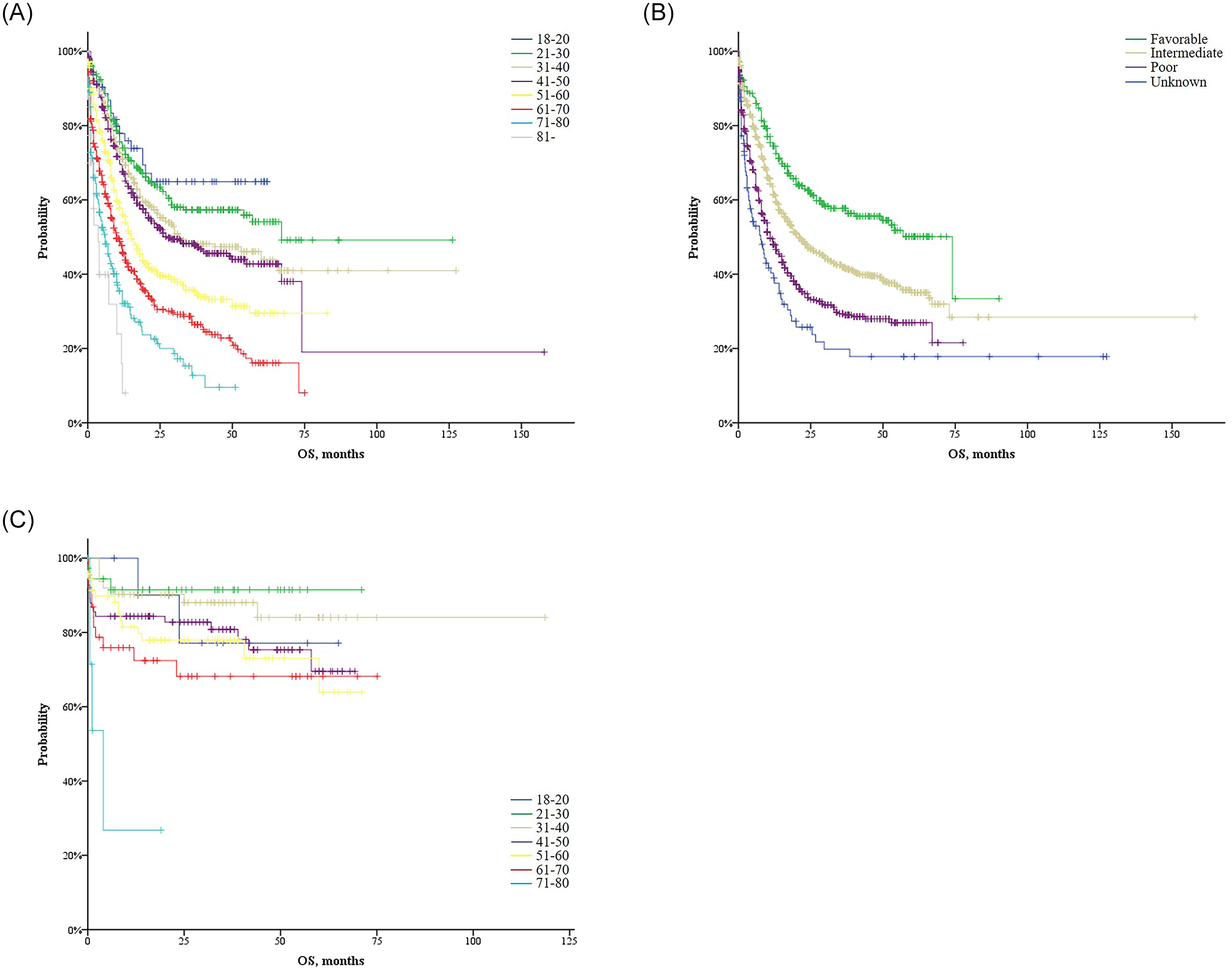

Supplement: S1 Fig — (TIF) [file pone.0251011.s001.tif]

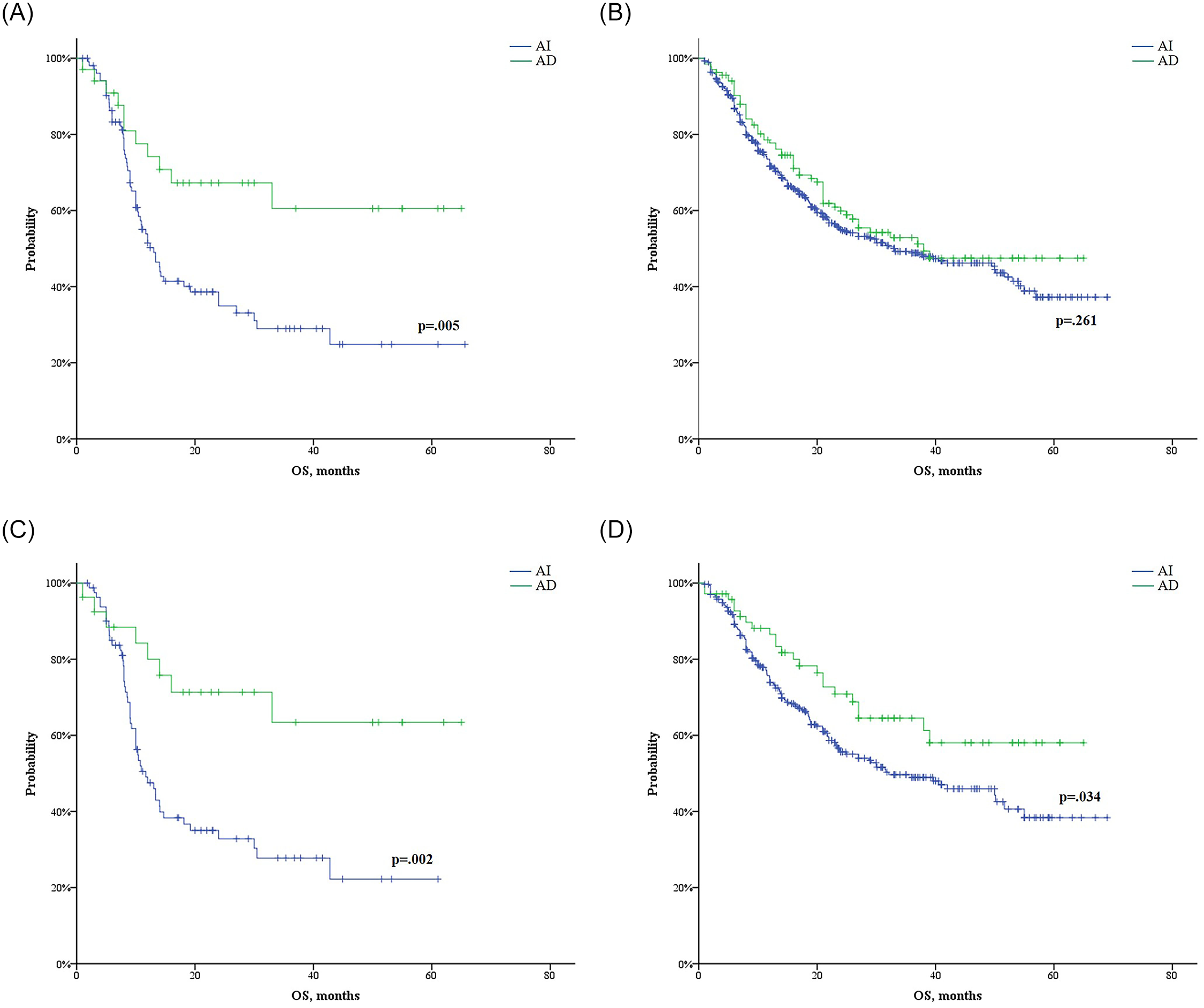

Supplement: S2 Fig — (TIF) [file pone.0251011.s002.tif]
