# Supplementary material for: Age and remission induction therapy for acute myeloid leukemia: An analysis of data from the Korean acute myeloid leukemia registry
Source: PLoS One. 2021 May 7;16(5):e0251011. doi: 10.1371/journal.pone.0251011 (PMC8104390; doi:10.1371/journal.pone.0251011)
Supplement: Attachment — Submitted filename: Response to Reviewers.docx [file pone.0251011.s003.docx]

Journal requirements:

When submitting your revision, we need you to address these additional requirements.

1. Please ensure that your manuscript meets PLOS ONE's style requirements, including those for file naming. The PLOS ONE style templates can be found at https://journals.plos.org/plosone/s/file?id=wjVg/PLOSOne_formatting_sample_main_body.pdf and https://journals.plos.org/plosone/s/file?id=ba62/PLOSOne_formatting_sample_title_authors_affiliations.pdf

🡪 We applied recommended PLOS ONE's style requirements to the manuscript.

2. Your ethics statement should only appear in the Methods section of your manuscript. If your ethics statement is written in any section besides the Methods, please move it to the Methods section and delete it from any other section. Please ensure that your ethics statement is included in your manuscript, as the ethics statement entered into the online submission form will not be published alongside your manuscript.

🡪 We moved ethics statement to method section from the end of manuscript.

[Note: HTML markup is below. Please do not edit.]

Reviewers' comments:

Reviewer's Responses to Questions

Comments to the Author

1. Is the manuscript technically sound, and do the data support the conclusions?

The manuscript must describe a technically sound piece of scientific research with data that supports the conclusions. Experiments must have been conducted rigorously, with appropriate controls, replication, and sample sizes. The conclusions must be drawn appropriately based on the data presented.

Reviewer #1: No

Reviewer #2: Yes

2. Has the statistical analysis been performed appropriately and rigorously?

Reviewer #1: Yes

Reviewer #2: Yes

3. Have the authors made all data underlying the findings in their manuscript fully available?

The PLOS Data policy requires authors to make all data underlying the findings described in their manuscript fully available without restriction, with rare exception (please refer to the Data Availability Statement in the manuscript PDF file). The data should be provided as part of the manuscript or its supporting information, or deposited to a public repository. For example, in addition to summary statistics, the data points behind means, medians and variance measures should be available. If there are restrictions on publicly sharing data—e.g. participant privacy or use of data from a third party—those must be specified.

Reviewer #1: No

Reviewer #2: Yes

4. Is the manuscript presented in an intelligible fashion and written in standard English?

PLOS ONE does not copyedit accepted manuscripts, so the language in submitted articles must be clear, correct, and unambiguous. Any typographical or grammatical errors should be corrected at revision, so please note any specific errors here.

Reviewer #1: Yes

Reviewer #2: Yes

5. Review Comments to the Author

Please use the space provided to explain your answers to the questions above. You may also include additional comments for the author, including concerns about dual publication, research ethics, or publication ethics. (Please upload your review as an attachment if it exceeds 20,000 characters)

Reviewer #1: The authors have performed a retrospective analysis aiming to evaluate clinical outcomes of AML patients.

The paper is written in a good but essential English, and it could be clear for readers.

However the analysis is based on a old era of AML, considering that molecular alterations are not considered in the analysis.

Patients were collected between 2007 and 2011, and the paper is not applicable to current era of this disease, and also references confirm that paper has been written and thought not recently (last references are from 2018).

The idea could be good, considering the number of patients (3011), but it should be updated to recent data and recent prognostic parameters.

To date, it should be completely re-designed and rewritten, and it must be rejected at this state.

🡪 I’d like to thank for the thorough review and inspection. Our study was based on the results of the registry program performed between 2007 and 2011 with the initiative of the AML/MDS working party of Korean Society of Hematology. As the reviewer pointed out, consistent molecular-based biological analysis was not possible because of the nature of a retrospective study and target population. New prospective cohort studies with molecular basis are needed.

Reviewer #2: The authors present a large national cohort of AML patients from South Korea. In this article, they focus mainly on the following aspects: Changes in prognosis and cytogenetics with age, with the data nicely presented by decade. Treatment of APL vs non-APL patients, and outcomes with daunorubicin/Ara-C vs idarubicin/Ara-C. The discussion is in depth and develops the data findings.

A few points could be addressed in the manuscript:

-How were the 500 or so patients who did not receive intensive chemotherapy treated? What was their outcome?

🡪 Most of patients who did not received intensive chemotherapy were reported to have been transferred to another hospital. They were likely to have had intensive chemotherapy at the hospital they moved to. Due to the limitation of a registry study, the exact outcome of these patients was not known. Some elderly patients were reported to have given up intensive chemotherapy and just received palliative care.

-Is the registry collecting functional status and comorbidity data as, as the authors acknowledge themselves, these may contribute to age differences in prognosis?

🡪 The performance status and comorbidities at the time of diagnosis are important to expect treatment outcome of acute leukemia. However, unified indexes for performance status and comorbidities were not applicable to this registry. Instead of these indicators, we focused on the patients who received induction chemotherapy, regarded as having qualified functional status.

6. PLOS authors have the option to publish the peer review history of their article (what does this mean?). If published, this will include your full peer review and any attached files.

If you choose “no”, your identity will remain anonymous but your review may still be made public.

Do you want your identity to be public for this peer review? For information about this choice, including consent withdrawal, please see our Privacy Policy.

Reviewer #1: No

Reviewer #2: Yes: Martine Extermann, MD, PhD
